# Debunking the myth: are soy isoflavones truly a public health concern?

**DOI:** 10.3389/fnut.2025.1608814

**Published:** 2025-07-15

**Authors:** Miguel López-Moreno, José Francisco López-Gil

**Affiliations:** ^1^Diet, Planetary Health and Performance, Faculty of Health Sciences, Universidad Francisco de Vitoria, Madrid, Spain; ^2^One Health Research Group, Universidad de Las Américas, Quito, Ecuador

**Keywords:** isoflavones, children, endocrine, hormones, soy, phytoestrogens

## 1 Introduction

Isoflavones are phytoestrogens, which are plant-derived compounds found in high concentrations in soy and other legumes, such as chickpeas and lentils. These molecules exhibit estrogen-like activity, which has been linked to both potential health benefits, such as reducing menopausal symptoms, improving bone health, and preventing hormone-related cancers, and concerns about their endocrine-disrupting effects (1, 2). Children, as a developing population, are considered particularly vulnerable to exposure to substances with endocrine activity due to their ongoing hormonal, metabolic and reproductive development (3). Despite the growing use of soy-based products in pediatric nutrition, especially in the form of infant formulas and plant-based alternatives, regulatory agencies have issued inconsistent recommendations, reflecting a lack of consensus on the interpretation of the available evidence. This study aims to analyze in detail the recommendations issued by the French Agency for Food, Environmental and Occupational Health & Safety (ANSES) regarding isoflavone consumption in children, evaluating the basis of these measures in light of broader scientific evidence, with a particular focus on studies conducted in humans.

## 2 ANSES recommendations

ANSES conducted a comprehensive review to assess the safety of isoflavones, analyzing numerous toxicological and epidemiological studies on isoflavone consumption and its potential effects on human health (4). As a result of this review, the ANSES defines the toxicological reference value (TRV) for daily isoflavone intake. For the general population, a TRV of 0.02 mg per kg of body weight per day was established. For vulnerable populations, which include children before puberty, women of reproductive age, and pregnant women, the TRV was halved to 0.01 mg per kg of body weight per day. ANSES revealed a substantial risk of exceeding these TRVs among individuals who consumed soy-based foods. Importantly, the ANSES report focuses on concerns about isoflavones and hormone-related effects but does not evaluate the benefits of soy, soy products, or isoflavones themselves. In Europe, risk and benefit assessments are handled separately, so ANSES's conclusions do not consider any potential positive effects of soy consumption. While these limits may appear conservative, the unilateral focus on potential risks without consideration of benefits warrants scrutiny.

## 3 Limitations of the animal studies

ANSES bases its concerns primarily on preclinical evidence derived from animal studies. Animal studies have historically formed the foundation for concerns about isoflavones as endocrine disruptors. These studies, while valuable for exploring biological mechanisms, face significant limitations when applied to human health assessments. Rodents have significantly less efficient phase II metabolism via glucuronidation, which leads to a reduced capacity for isoflavone conjugation and results in markedly higher plasma concentrations of unconjugated genistein than humans do (5, 6). These metabolic differences raise concerns about the reliability of rodent models for evaluating the effects of isoflavones on human tissues, particularly estrogen-sensitive breast tissue. Many animal studies use doses of isoflavones that far exceed typical human dietary intake, which can exaggerate effects not observed at realistic consumption levels (6). Additionally, the routes of administration in animal experiments, such as injections or gavage, bypass the digestive processes that occur in humans, altering the bioavailability and activity of the compounds (7, 8). Furthermore, the gut microbiota plays a crucial role in metabolizing isoflavones into active metabolites such as equol, and this microbiota composition varies significantly between species, influencing outcomes (9). These factors collectively limit the applicability of animal data to human contexts and underscore the need for caution when extrapolating findings across species.

## 4 Human studies: a reassuring perspective

In contrast to the animal data, evidence from human studies offers a far more reassuring picture regarding isoflavone exposure. Systematic reviews and meta-analyses have demonstrated no significant association between soy-based infant diets and the onset of puberty in humans, including precocious puberty or age at menarche (10). These findings contradict earlier concerns based on animal studies, which suggested that phytoestrogens in soy formulas could disrupt hormonal development (11–13). Human data consistently show that soy consumption during infancy does not pose a risk to normal pubertal timing. The disparity between ANSES's approach and that of other international health authorities highlights a pressing need for harmonized, evidence-based guidelines.

Long-term evaluations of children fed soy protein formulas have shown no evidence of estrogen-like hormonal effects. Bone metabolism markers remain within normal ranges, and there are no signs of precocious puberty or gynecomastia in children consuming soy-based diets (14). Although infants fed soy formulas present higher levels of isoflavones than do those fed cow milk formula or breast milk, these concentrations do not correlate with hormonal changes, indicating minimal biological impact (15).

Further longitudinal and cross-sectional studies confirmed that pubertal development trajectories are unaffected by soy consumption. For example, breast bud diameter and hormone concentrations in girls fed soy formula are comparable to those in those fed cow milk formula, and no differences in estradiol levels are detectable in boys between these groups (16). Additionally, adolescent girls with varying levels of soy intake show no differences in age at menarche, reinforcing the conclusion that soy-based diets do not influence pubertal timing or hormonal development (17). Although the majority of human studies report no adverse effects of soy on pubertal development, many are observational and subject to potential confounding. Nevertheless, the consistency of findings across diverse populations strengthens the reliability of the overall conclusion.

## 5 Precautionary principles vs. evidence-based recommendations

ANSES's restrictive stance on isoflavones, and consequently soy-based products, reflects an application of the precautionary principle—a strategy aimed at minimizing potential risks when scientific uncertainty exists. While this approach prioritizes safety, it may inadvertently disregard substantial evidence supporting the potential benefits of soy products in humans.

Organizations such as the European Food Safety Authority (EFSA) have evaluated the safety of isoflavones, particularly as food supplements for postmenopausal women, and found no evidence of harm to the mammary glands, uterus, or thyroid function at typical supplementation levels (18). Canada's Dietary Guidelines include soy in its dietary guidelines as a healthy food, indicating a general acceptance of soy consumption as part of a balanced diet (19). The Australian Dietary Guidelines include soy products as part of a healthy diet for children and adolescents (20). The U.S. National Institutes of Health considers soy a safe food for most people, suggesting a broad consensus on its safety in the general population (21). The Scientific Report of the 2025 Dietary Guidelines Advisory Committee highlights the inclusion of soy products as part of a healthy diet during childhood, emphasizing their role in providing high-quality protein and essential micronutrients critical for growth and development. Furthermore, in adults, replacing processed or unprocessed red meat with plant-based protein sources, including soy, is associated with a reduced risk of cardiovascular disease morbidity (22).

## 6 Conclusion

The debate over soy isoflavones exemplifies the complexities of translating scientific research into public health policy. While animal studies have raised valid concerns about their endocrine activity, robust human data consistently refute their classification as endocrine disruptors or harmful to health. Moving forward, dietary recommendations should prioritize evidence-based approaches that consider both safety and nutritional value. Future research should aim to strengthen causal inference through studies with detailed dietary assessments and long-term follow-up, as well as mechanistic studies that clarify the biological pathways through which isoflavones exert their effects.

Ultimately, fostering collaboration among regulatory agencies and researchers will ensure that public health policies are informed by comprehensive scientific understanding rather than isolated findings. Soy foods remain a safe and valuable component of human diets when consumed responsibly, a conclusion supported by decades of research across diverse populations. Rather than focusing solely on potential risks, public health policy should also recognize and embrace the well-established benefits of soy consumption in the context of overall dietary quality and sustainability.

## References

[1] Jargin SV. Soy and phytoestrogens: possible side effects. Ger Med Sci. (2014) 12:Doc18. 10.3205/000203 25587246 PMC4270274

[2] Gómez-Zorita S González-Arceo M Fernández-Quintela A Eseberri I Trepiana J Portillo MP. Scientific evidence supporting the beneficial effects of isoflavones on human health. Nutrients. (2020) 12:3853. 10.3390/nu12123853 33348600 PMC7766685

[3] Messina M Rogero MM Fisberg M Waitzberg D. Health impact of childhood and adolescent soy consumption. Nutr Rev. (2017) 75:500–15. 10.1093/nutrit/nux016 28838083

[4] ANSES-National Agency for Food Environmental and Occupational Health Safety. Avoiding Isoflavones in Catering Menus. Available online at: https://www.anses.fr/fr/content/eviter-les-isoflavones-dans-les-menus-des-restaurations-collectives (Accessed April 4, 2025).

[5] Gu L House SE Prior RL Fang N Ronis MJJ Clarkson TB. Metabolic phenotype of isoflavones differ among female rats, pigs, monkeys, and women. J Nutr. (2006) 136:1215–21. 10.1093/jn/136.5.1215 16614407

[6] Setchell KDR Brown NM Zhao X Lindley SL Heubi JE King EC. Soy isoflavone phase II metabolism differs between rodents and humans: implications for the effect on breast cancer risk. Am J Clin Nutr. (2011) 94:1284–94. 10.3945/ajcn.111.019638 21955647 PMC3192476

[7] Caceres S Silvan G Martinez-Fernandez L Illera MJ Millan P Monsalve B. The effects of isoflavones on androgens and glucocorticoids during puberty on male Wistar rats. Reprod Domest Anim. (2014) 49:611–7. 10.1111/rda.12335 24930378

[8] Milošević VL Severs WB Ristić NM Manojlović-Stojanoski MN Popovska-Perčinić FV Šošić-Jurjević BT. Soy isoflavone effects on the adrenal glands of orchidectomized adult male rats: a comprehensive histological and hormonal study. Histol Histopathol. (2018) 33:843–57. 10.14670/HH-11-984 29528085

[9] Tsuchihashi R Sakamoto S Kodera M Nohara T Kinjo J. Microbial metabolism of soy isoflavones by human intestinal bacterial strains. J Nat Med. (2008) 62:456–60. 10.1007/s11418-008-0271-y 18648905

[10] Messina M Mejia SB Cassidy A Duncan A Kurzer M Nagato C. Neither soyfoods nor isoflavones warrant classification as endocrine disruptors: a technical review of the observational and clinical data. Crit Rev Food Sci Nutr. (2022) 62:5824–85. 10.1080/10408398.2021.1895054 33775173

[11] Glover A Assinder SJ. Acute exposure of adult male rats to dietary phytoestrogens reduces fecundity and alters epididymal steroid hormone receptor expression. J Endocrinol. (2006) 189:565–73. 10.1677/joe.1.06709 16731787

[12] Caceres S Crespo B Alonso-Diez A de Andrés PJ Millan P Silván G. Long-term exposure to isoflavones alters the hormonal steroid homeostasis-impairing reproductive function in adult male Wistar rats. Nutrients. (2023) 15:1261. 10.3390/nu15051261 36904260 PMC10005734

[13] Weber KS Setchell KDR Stocco DM Lephart ED. Dietary soy-phytoestrogens decrease testosterone levels and prostate weight without altering LH, prostate 5alpha-reductase or testicular steroidogenic acute regulatory peptide levels in adult male Sprague-Dawley rats. J Endocrinol. (2001) 170:591–9. 10.1677/joe.0.1700591 11524239

[14] Giampietro PG Bruno G Furcolo G Casati A Brunetti E Spadoni GL. Soy protein formulas in children: no hormonal effects in long-term feeding. J Pediatr Endocrinol Metab. (2004) 17:191–6. 10.1515/JPEM.2004.17.2.191 15055353

[15] Cao Y Calafat AM Doerge DR Umbach DM Bernbaum JC Twaddle NC. Isoflavones in urine, saliva and blood of infants - data from a pilot study on the estrogenic activity of soy formula. J Expo Sci Environ Epidemiol. (2008) 19:223. 10.1038/JES.2008.44 18665197 PMC2630504

[16] Adgent MA Umbach DM Zemel BS Kelly A Schall JI Ford EG. A longitudinal study of estrogen-responsive tissues and hormone concentrations in infants fed soy formula. J Clin Endocrinol Metab. (2018) 103:1899–909. 10.1210/jc.2017-02249 29506126 PMC6456922

[17] Segovia-Siapco G Pribis P Messina M Oda K Sabaté J. Is soy intake related to age at onset of menarche? A cross-sectional study among adolescents with a wide range of soy food consumption. Nutr J. (2014) 13:54. 10.1186/1475-2891-13-54 24889551 PMC4051381

[18] Risk assessment for peri- and post-menopausal women taking food supplements containing isolated isoflavones. EFSA J. (2015) 13:4246. 10.2903/j.efsa.2015.4246

[19] Health Canada. Canada's Dietary Guidelines - Canada's Food Guide. Available online at: https://food-guide.canada.ca/en/guidelines/ (Accessed April 4, 2025).

[20] Australian Government. The Australian Dietary Guidelines | Australian Government Department of Health and Aged Care. Available online at: https://www.health.gov.au/resources/publications/the-australian-dietary-guidelines?language=en (Accessed April 4, 2025).

[21] National Center for Complementary and Integrative Health. Soy: Usefulness and Safety | NCCIH. Available online at: https://www.nccih.nih.gov/health/soy (Accessed April 4, 2025).

[22] 2025 Dietary Guidelines Advisory Committee. Scientific Report of the 2025 Dietary Guidelines Advisory Committee: Advisory Report to the Secretary of Health and Human Services and Secretary of Agriculture (2024). 10.52570/DGAC2025

